# Prognostic Markers Associated with Short-Term Mortality in Dogs Hospitalised with Acute Pancreatitis: A Retrospective Study of 192 Cases

**DOI:** 10.3390/ani16121854

**Published:** 2026-06-16

**Authors:** Casandra Juárez Sarrión, Iván Rodríguez Armas, Ana Torrano Guillamón, Jorge Castro López, Carolina Arenas Bermejo

**Affiliations:** 1 AniCura Valencia Sur Veterinary Hospital, 46460 Silla, Valencia, Spain; 2 AniCura Indautxu Veterinary Hospital, 48950 Bilbao, Bizkaia, Spain

**Keywords:** acute pancreatitis, dog, prognosis, biomarkers, alkaline phosphatase, mortality, creatinine, bilirubin

## Abstract

Objectives: To describe the clinical, clinicopathological and ultrasonographic characteristics of dogs with an acute pancreatitis (AP) and to identify prognostic markers associated with short-term mortality in a referral population. Methods: This retrospective observational study included 192 dogs hospitalised with AP at a referral centre in Spain between 2021 and 2024. Dogs were classified as survivors or non-survivors based on survival to hospital discharge. Signalment, clinical findings, clinicopathological and ultrasonographic variables, comorbidities, treatments and duration of hospitalisation were recorded. Variables associated with outcome in univariable analyses were entered into a multivariable logistic regression model to identify independent predictors of death. Results: Of the 192 dogs, 141 (73.4%) survived and 51 (26.6%) died or were euthanised during hospitalisation. Non-survivors had higher serum creatinine, total bilirubin and alkaline phosphatase (ALKP) concentrations than survivors. In multivariable analysis, increased serum creatinine (odds ratio [OR] 2.33; 95% confidence interval [CI] 1.32–4.11), total bilirubin (OR 1.42; 95% CI 1.03–2.06) and ALKP (OR 1.52; 95% CI 1.07–2.16) were independently associated with an increased risk of short-term mortality. Ultrasonographic abnormalities compatible with AP were identified in 121 dogs (63%), but were not independently associated with short-term mortality. Breed, age, sex, body weight, comorbidities and treatments were not associated with outcome. Clinical Significance: Measurement of serum creatinine, total bilirubin and ALKP concentrations at presentation may help identify dogs with AP at increased risk of death. Early recognition of high-risk patients may assist clinical decision-making, guide the intensity of monitoring, and facilitate communication with owners.

## Simple Summary

Acute pancreatitis is a common and potentially severe disease in dogs, but early identification of patients at higher risk of death remains challenging. This retrospective study evaluated 192 dogs hospitalised with acute pancreatitis and identified serum creatinine, total bilirubin and alkaline phosphatase concentrations measured at admission as prognostic markers associated with short-term mortality. While creatinine and bilirubin have previously been linked to a poor outcome, the association between alkaline phosphatase and mortality may represent a novel finding in dogs with acute pancreatitis. These results may help clinicians identify high-risk patients earlier, guide monitoring intensity and improve communication with owners.

## 1. Introduction

Acute pancreatitis (AP) is relatively common in dogs [1,2,3,4]. To date, the diagnosis of AP and the assessment of its severity remain challenging [1,2,3,4]. The gold standard in diagnosis and classification of pancreatitis in dogs is histopathological evaluation; however, this is rarely performed given its invasive nature [1,2]. Clinical diagnosis is usually based on clinical signs and physical examination findings, ultrasound findings and laboratory tests, including specific pancreatic lipase tests [1,2].

Pancreatitis presents with a broad clinical spectrum, ranging from mild to severe disease.

Mild pancreatitis, which is characterised by moderate clinical signs, generally results in full recovery with appropriate medical treatment [5,6]. Nevertheless, in severe pancreatitis, acute pancreatic necrosis results in more severe clinical signs and multisystem complications such as systemic inflammatory response syndrome (SIRS), multiple organ dysfunction syndrome, or disseminated intravascular coagulation [5,6]. In human medicine, early identification of severe AP is considered essential for identifying patients at a higher risk of disease progression and initiating appropriate treatment as early as possible, while trying to decrease the death rate. In veterinary medicine, clinical severity indices have been developed for dogs with acute pancreatitis (AP) [4,5,6,7].

Previous studies have identified azotaemia, hyperphosphatemia, hyponatraemia, hypocalcaemia, increased total bilirubin, thrombocytopenia, coagulation abnormalities, ascites, SIRS, acute respiratory distress syndrome (ARDS) and acute kidney injury (AKI) as negative prognostic markers in dogs with AP [7,8].

Despite increased knowledge about AP, the death rate in dogs remains high, ranging from 23% to 40%, contrasting with the 5% to 15% death rate reported in human medicine [5,9]. Consequently, the severity assessment of AP and the identification of prognostic factors in dogs remain a major challenge to identify patients at an increased risk of complications [3].

This study aimed to describe the clinical signs, clinicopathological findings, and ultrasound findings of a population of dogs diagnosed with pancreatitis with an AP in a referral hospital and to identify short-term AP-related mortality, allowing rapid identification of those patients at increased risk of mortality.

## 2. Materials and Methods

In this retrospective observational study, electronic medical records were searched between 2021 and 2024 to identify dogs diagnosed with AP. Dogs were eligible for inclusion if the medical record was complete and contained detailed clinical history, haematology, serum biochemistry, urinalysis, abdominal ultrasound and specific canine pancreatic lipase (Spec cPL) or activity of 1,2-o-dilauryl-rac-glycero-3-glutaric acid-(6’-methylresorufin) ester (DGGR) performed. All clinicopathological variables, including Spec cPL and DGGR-based lipase activity, were recorded from the samples obtained at hospital admission. DGGR-based lipase activity and Spec cPL were measured at an external laboratory (IDEXX Laboratories, Barcelona, Spain), and the cut-off value was applied according to the reference interval provided by the laboratory.

Abdominal ultrasound examinations were performed by board-certified specialists or by residents or interns in specialty training under their direct supervision. All included cases required a complete ultrasound report documenting the evaluation of the pancreas; cases with incomplete data were excluded. A Canon Aplio i600 ultrasound machine (Canon Medical Systems Corporation, Otawara, Japan) with convex (9–10.8 MHz) and linear (12–18 MHz) probes was used.

The diagnosis of AP was based on the following inclusion criteria: (1) ≥2 compatible clinical signs: vomiting, diarrhoea, hyporexia/anorexia, abdominal pain and lethargy for <7 days; (2) ≥2 compatible ultrasound findings: hypoechoic pancreatic parenchyma, enlarged pancreas, irregular pancreatic margins, localised peritoneal free fluid, hyperechoic peripancreatic peritoneum and pancreatic hypoechoic stripes; (3) spec cPL concentrations > 400 μg/L or DGGR > 200 U/L.

Patients fulfilling all three criteria were considered compatible with AP. Patients meeting two of the three criteria (compatible clinical signs plus ultrasound findings or increased Spec cPL or DGGR) were classified as highly suspected of having AP.

Dog features, including sex, breed, age, and weight, were recorded. Presenting complaints, physical examination, results of diagnostic tests performed at the time of hospital admission, including haematology, serum biochemistry, urinalysis, abdominal ultrasound, and comorbidities, treatments received and hospitalisation time were recorded.

Patients were classified into survivors and non-survivors based on whether or not they survived the hospitalisation discharge.

The data of signalment, diet, clinical signs, laboratory and ultrasonographic findings, comorbidities, treatments administered, and length of hospitalisation were compared between the two groups.

This retrospective study was conducted using data obtained from the hospital records. No ethical approval or owner consent was required according to the institutional guidelines.

### Statistical Analysis

All statistical analyses were performed using Minitab 21 (Minitab LLC, State College, PA, USA). Continuous variables were tested for normality using visual inspection and the Kolmogorov–Smirnov test. As most variables were not normally distributed, data were summarised as medians and interquartile ranges (IQR) and compared between survivors and non-survivors using the Mann–Whitney U test adjusted for ties. Categorical variables were compared using Fisher’s exact test. Variables with a *p* value < 0.20 in univariable analyses were included as candidates in a binary logistic regression model to identify independent predictors of death. For the analysis of prognostic markers, only deaths directly attributable to acute pancreatitis were considered, while deaths from unrelated causes were excluded from this analysis. Continuous variables showing skewed distributions were log-transformed prior to their inclusion in the model. Backward stepwise elimination was applied, and significance was set at *p* < 0.05. Correlations between the selected biochemical parameters and duration of hospitalisation were assessed using Spearman’s rank correlation coefficient.

## 3. Results

From the medical records, 207 hospitalised dogs with AP were identified. Of these, 15 dogs were excluded due to a lack of a complete ultrasound report and/or images (10 dogs) or insufficient medical data (5 dogs). Finally, a total of 192 dogs met the inclusion criteria and they were included in this retrospective study. A total of 112 dogs (58.3%) were included in the AP group and 80 (41.7%) as high suspicion of AP.

There were 92 males (44 intact and 48 neutered) and 100 females (32 intact and 68 spayed) dogs. The median age was 9 years and median weight was 10.7 kg.

In this study, 44 breeds were included. The most common were mixed-breed (*n* = 66), Terriers (*n* = 38), Maltese (*n* = 8), Miniature Schnauzers (*n* = 8), Pomeranians (*n* = 7), Chihuahuas (*n* = 6), Golden Retrievers (*n* = 6), Toy Poodles (*n* = 5), Dachshunds (*n* = 5), Jack Russell Terriers (*n* = 5), French Bulldogs (*n* = 4), Beagles (*n* = 4), Pinschers (*n* = 4), Cocker Spaniels (*n* = 3), Labrador Retrievers (*n* = 3), Shih Tzus (*n* = 3), American Bullies (*n* = 3), Pugs (*n* = 3), Samoyeds (*n* = 3),West Highland White Terriers (*n* = 3), Boxers (*n* = 2), Rottweilers (*n* = 2), Siberian Huskies (*n* = 2), Spanish Mastiffs (*n* = 2) and one of each from 20 other breeds.

All included dogs presented with at least two clinical signs compatible with AP. Most frequent clinical signs at presentation were lethargy in all dogs (100%), abdominal pain in 183 dogs (95.3%), hyporexia/anorexia in 182 (94.8%), vomiting in 136 dogs (70.8%) and diarrhoea in 133 dogs (69.3%).

The most common laboratory findings were monocytosis in 99 dogs (51.6%), anaemia in 98 dogs (51%), hypertriglyceridemia in 97 dogs (50.5%) and neutrophilia in 95 dogs (49.5%).

Ultrasonographic abnormalities compatible with AP were identified in 121/192 dogs (63%). In all these cases, at least two compatible ultrasonographic findings were present, fulfilling the ultrasonographic criteria for acute pancreatitis. The most frequent ultrasound findings were focal free fluid in 96/121 dogs (79.3%), hyperechoic peripancreatic peritoneum in 81/121 dogs (66.9%), enlarged pancreas in 73/121 dogs (60.3%) and pancreatic hypoechoic stripes in 52/121 dogs (42.9%).

Comorbidities were detected in 173/192 dogs (90.1%) and were classified into the following groups: infectious diseases in 33/192 dogs (17.1%), gastrointestinal diseases in 26/192 dogs (13.5%), liver diseases in 24 dogs (12.5%), immune-mediated diseases in 19 dogs (9.9%), endocrinopathies in 17/192 dogs (8.9%), renal diseases in 16/192 dogs (8.4%), neoplasia in 16/192 dogs (8.4%), cardiorespiratory diseases in 10/192 dogs (5.2%), neurological diseases in 6/192 dogs (3.1%) and others in 6/192 dogs (3.1%).

A total of 54/192 dogs (28.1%) received treatments considered possible risk factors for the AP: corticosteroids in 25/192 dogs (13%), meglumine antimoniate in 11/192 dogs (5.7%), non-steroidal anti-inflammatory drugs in 10/192 dogs (5.2%), phenobarbital in 6/192 dogs (3.1%) and furosemide in 2/192 dogs (1%).

**Figure 1:**
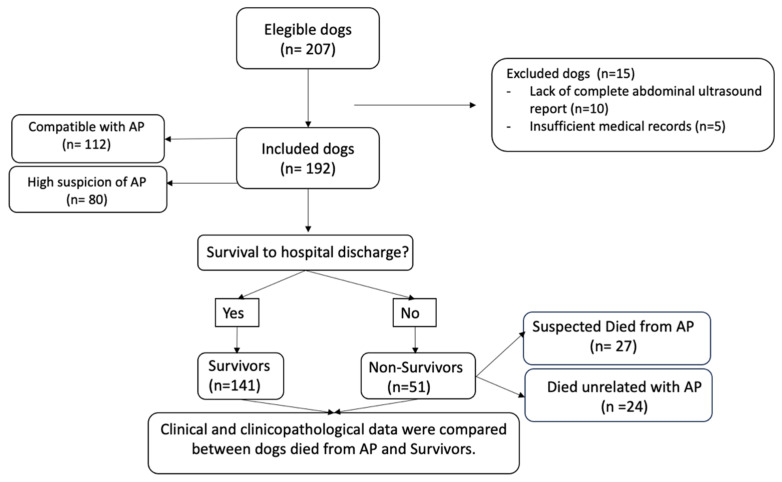
Flowchart illustrating the selection of dogs included in the study.

Of the 192 dogs, 141 survived (73.4%) and 51 died/were euthanised (26.5%). In total 27 dogs (14%) died from causes directly attributable to AP; of these, 15 dogs (55.55%) were euthanised due to unfavourable disease progression and clinical deterioration. A total of 24 dogs (12.5%) died from causes not directly attributable to AP. The analysis of prognostic markers was performed considering only the deaths directly attributable to acute pancreatitis. The causes of death not directly attributable to AP are shown in Table 1. The flowchart illustrating case selection and final study population is shown in Figure 1.

Regarding clinicopathological findings, non-surviving dogs had significantly higher creatinine (*p* = 0.008), phosphorus (*p* = 0.035), alanine aminotransferase (ALT) (*p* = 0.005), alkaline phosphatase (ALKP) (*p* = 0.002), gamma-glutamyl transferase (GGT) (*p* = 0.016) and total bilirubin (*p* = 0.004) concentrations than survivors (Table 2). Univariable analyses conducted both on the entire cohort and on the subset with confirmed AP yielded similar significant predictors. Owing to the consistency of these results, both groups were grouped together and the multivariable analysis was performed on the full population to enhance statistical power. After multivariate analysis, only increased creatinine (*p* = 0.003; OR 2.333; CI 1.323–4.111), total bilirubin (*p* = 0.036; OR 1.424; CI 1.025–2.058) and ALKP (*p* = 0.021; OR 1.518; CI 1.065–2.163) remained independent predictors of poor prognosis (Table 3).

Spec cPL concentrations were available in 54 dogs (28.1%), of which 53 (98.1%) had values > 400 μg/L. DGGR-based lipase activity was measured in 138 dogs (71.9%), with 136 (98.6%) showing values > 200 U/L. Median Spec cPL concentration was 1019 μg/L (IQR 649–1537), while median DGGR-based lipase activity was 595.5 U/L (IQR 329.5–1297).

Because Spec cPL and DGGR-based lipase activity were measured using different analytical scales, both variables were log-transformed and standardised prior to additional exploratory analysis. No significant differences were identified between both assays.

No significant differences were observed between survivors and non-survivors for either assay.

The median hospitalisation time was 4 days (IQR 3–6). There was no significant difference in median hospitalisation time between the survivors and non-survivors. Higher ALKP (rs = 0.12; *p* = 0.10), total bilirubin (rs = 0.07; *p* = 0.36) and creatinine (rs = 0.10; *p* = 0.17) concentrations were not associated with longer hospitalisation time.

Breed, age, sex, neutering, body weight, comorbidities and treatments received had no significant association with prognosis.

Similarly, no significant differences in ALKP concentrations were observed between dogs receiving glucocorticoids and those not receiving these treatments (*p* = 0.98).

## 4. Discussion

This study describes clinical and clinicopathological features of a population of dogs with AP in a referral hospital. Although similar studies may have been published, the canine population evaluated in this study differs, and therefore the data presented here may provide valuable and novel insights for veterinary clinicians. In Spain, pet insurance coverage remains uncommon, and financial limitations may affect owner acceptance of diagnostic procedures, prolonged hospitalisation or intensive care monitoring, which may affect the outcome. In addition, differences in regional clinical practices may influence the use of drugs not routinely administered in other countries, such as meglumine antimoniate, which has previously been associated with pancreatitis [2].

Several dogs in the present study had also received medications previously described as possible risk factors for AP, including corticosteroids [3]. However, the association between glucocorticoid administration and pancreatitis in dogs remains controversial in the veterinary literature, and causality cannot be established based on the present retrospective study [3]. In the present study, we did not identify any association between the administration of these medications and AP-related mortality.

Clinical signs, ultrasonographic findings and abnormal Spec cPL or DGGR results were used as inclusion criteria, as described in other studies [3,4,5,6,7,8,9,10]. Both Spec cPL and DGGR results were included as criteria for laboratory diagnosis, as a high agreement between both tests has been previously demonstrated [1,2,10]. The sensitivity and specificity of ultrasonographic diagnosis of canine AP is highly variable depending on the studies, and there may be dogs with AP that do not present compatible ultrasonographic abnormalities at the time of presentation, which may be observed later [1,2,10,11]. Therefore, it was decided to also include dogs without significant ultrasonographic alterations at presentation.

It should also be noted that increased pancreatic lipase activity is not specific for acute pancreatitis in critically ill dogs [12]. Previous studies have shown that hyperlipasemia may occur in a variety of non-pancreatic conditions, including renal, endocrine and immune-mediated diseases, and may also be associated with worse outcomes independent of pancreatitis [12]. Therefore, reliance on pancreatic lipase activity alone may lead to overdiagnosis of AP. For this reason, according to current diagnostic recommendations, pancreatic lipase assays were interpreted in combination with clinical signs and ultrasonographic findings in order to improve diagnostic accuracy.

The age, weight and breeds of our study population are consistent with studies previously described [2,3,4,5,6,7,8,9,10,11].

The median hospitalisation time was four days, similar to that previously reported [4,5]. Although no significant differences were observed in the median hospitalisation time between the survivors and non-survivors, and higher serum concentrations of ALKP, total bilirubin and creatinine were not associated with longer hospitalisation time, it is important to note that 55.5% of non-survivor patients in this study were euthanised due to disease progression and clinical deterioration, which may have affected the results.

The mortality rate directly attributable to AP in our study was 14%, lower than previously described (23% to 40%) [5,9,13]. However, different inclusion criteria, unspecified treatments and population differences make it difficult to compare results.

In this study, we identified three independent risk factors for short-term death in dogs with AP: elevated serum creatinine levels, hyperbilirubinemia and elevated serum ALKP levels.

Azotemia has been described as a negative prognostic marker in dogs with AP [5,7,9]. Furthermore, the development of AKI has also been associated with a poorer prognosis in dogs with AP [14]. This is consistent with our results, where elevated creatinine levels were associated with increased mortality. During AP, AKI can occur due to various mechanisms previously described, including events that lead to renal microcirculation damage, which could exacerbate ischemia and hypovolemia, thus worsening kidney damage [14]. In human medicine, various studies also have shown that AKI, besides being one of the most common complications of AP, is associated with poor outcomes [14].

In agreement with previous studies, our results identified total bilirubin concentration as a prognostic marker in dogs with acute pancreatitis. Guglielmini et al. (2022) demonstrated that serum bilirubin was an independent predictor of short-term mortality in affected dogs [13]. Similarly, in our study, total bilirubin concentrations were significantly higher in non-survivors compared with survivors, supporting the association between hyperbilirubinemia and a poorer outcome in dogs with AP.

In human medicine, elevated serum ALKP concentrations have been associated with disease progression and increased mortality in patients with AP. Potential mechanisms for elevated ALKP include cholestasis secondary to pancreatic inflammation, extrahepatic bile duct compression, hepatobiliary injury and systemic inflammatory responses [15]. Our findings might be consistent with these observations, suggesting that a similar pathophysiologic mechanism may occur in dogs. Interpretation of ALKP in dogs, however, warrants caution, as canine ALKP includes a corticosteroid-induced isoenzyme not present in humans. Therefore, increased ALKP activity may be induced by prior glucocorticoid administration. In order to explore this possibility, ALKP activity was compared between dogs receiving glucocorticoids and those not receiving these treatments, and no significant differences were identified (*p* = 0.98), suggesting that corticosteroid administration was unlikely to have significantly influenced ALKP values in this population.

Interestingly, although serum ALKP activity has been commonly included among the clinicopathological variables evaluated in previous veterinary studies of canine AP, to our knowledge, it has not previously been identified as an independent prognostic marker associated with mortality [13]. Therefore, this may represent a novel finding and may provide a basis for future veterinary studies specifically evaluating ALKP as an independent prognostic indicator in dogs with AP, similarly to observations previously reported in human medicine.

Finally, while both bilirubin and ALKP activity may be associated with cholestasis, they likely reflect different and not entirely overlapping pathophysiological processes. Hyperbilirubinemia may result from a combination of mechanisms, including impaired bile excretion, hepatocellular dysfunction or hemolysis. In contrast, increased ALKP activity, although also associated with cholestasis, may be influenced by additional factors such as systemic inflammation, stress-related enzyme induction, or concurrent treatments.

Consequently, increased ALKP activity in dogs with AP may not reflect pancreatitis-specific injury alone, but rather a combination of cholestasis, inflammatory and systemic disease processes associated with a more severe illness. Therefore, ALKP elevation may represent a broader systemic response rather than an isolated hepatobiliary dysfunction, which could explain its independent association with mortality in this study.

From a clinical perspective, identification of these variables at admission may assist in early risk stratification, the selection of patients requiring closer monitoring or intensive care, and more informed discussions with owners regarding prognosis.

Our study has several limitations, most of them inherent to its retrospective design. To begin with, some variables previously associated with mortality in AP may have been inadvertently excluded because they were not available in a sufficient number of cases to ensure adequate statistical power. Examples include blood gas analysis, ionised calcium, or thromboelastography. In addition, treatment protocols were not standardised, which could have influenced patient outcomes. Furthermore, information regarding corticosteroid type, dosage and treatment duration was not consistently available and could therefore not be further analysed.

Histopathology was also not available. Although it is considered the gold standard for pancreatitis diagnosis, it is rarely performed in clinical settings because most affected dogs are unstable, and the anaesthesia or invasive procedures may worsen their condition. No post-mortem examinations were performed, as these are not routinely pursued in clinical practice, require additional owner consent and incur additional costs that are often declined, particularly in populations with a low prevalence of pet insurance.

Most dogs in the study had comorbidities, which may have affected clinical signs, laboratory parameters, mortality rate, and hospitalisation time. Nonetheless, it is worth noting that the statistical analysis found no association between concurrent conditions and either short-term mortality or hospitalisation duration in the dogs included.

Classification of AP-related death was based on clinician assessment and retrospective medical record review; however, some degree of misclassification cannot be excluded. Moreover, the retrospective nature of the study did not always allow definitive distinction between pre-existing comorbidities and complications secondary to AP and in some cases, this distinction was particularly challenging. For example, although associative immunemiated hemolytic anaemia (IMHA) and/or thrombocytopenia are potential complications of pancreatitis, eight dogs were already diagnosed with immune-mediated conditions prior to presentation, so the cause of death was classified as non-related to AP. Similarly, although sepsis may occur as a complication of AP, the two dogs in which sepsis was the cause of death and that were classified within the non-AP-related death group had alternative sources of infection identified at presentation (post-operative pyometra and septic arthritis, respectively), making a direct causal relationship between sepsis and AP less likely. One dog also died due to hematogenous pneumonia. Although respiratory complications may occur secondary to AP (most commonly aspiration pneumonia or ARDS) this dog was included in the non-AP-related death group because its primary clinical sign at presentation was dyspnea.

Also, the inclusion of euthanised dogs may represent another source of bias, as the clinician’s assessment of the patient’s clinical or clinicopathological status during hospitalisation could have influenced the caregiver’s decision to opt for euthanasia. Similarly, misclassification of the outcome (i.e., erroneously attributing death to AP progression) may also have occurred.

Finally, the study was carried out in a referral hospital in Spain, which may limit extrapolation of these findings to primary care populations or other geographic locations.

## 5. Conclusions

In this study population, elevated serum creatinine, total bilirubin and ALKP concentrations were associated with an increased risk of short-term mortality in dogs with AP. While the association of creatinine and bilirubin with the outcome has been previously reported, the identification of ALKP as a prognostic marker may represent a novel finding in this population.

Assessment of these parameters may be useful for identifying patients with poorer prognosis that need more intensive monitoring, helping to identify and treat possible complications earlier.

## Figures and Tables

**Table 1:** Causes of death/euthanasia not directly attributable to AP. * UTI: urinary tract infection. OHE: ovariohysterectomy. IMHA: immune-mediated hemolytic anaemia.

Death Cause	Group	Number of Dogs	Concurrent Disease at the Time Pancreatitis Was Diagnosed
Evans syndrome	AP	2	Evans syndrome
Pyelonephritis	AP	1	UTI *
Congestive heart failure	AP	3	Mitral valve disease
Sepsis	AP	2	OHE due to pyometra Septic arthritis
Non-associative IMHA	High suspicion of AP	1	IMHA
Non-associative IMHA	AP	5	IMHA
Hematogenous pneumonia	High suspicion of AP	1	Hematogenous pneumonia
Protein-losing nephropathy	AP	2	Leishmania
Neoplasia	AP	6	Urothelial carcinoma, lymphoma, histiocytic sarcoma, cholangiocarcinoma
Cyca-induced toxic hepatitis	AP	1	Cyca-induced toxic hepatitis

**Table 2:** Clinical, ultrasound findings, comorbidities and laboratory data for the total study cohort and comparisons between dogs died from AP and survivors.

Parameter	Died from AP (*n* = 27)	Survivors (*n* = 141)	*p*
	Median	Median	
Signalment			
Age (years)	11 (IQR 8–13)	9 (IQR 6–12)	0.17
Weight (kg)	9.4 (IQR 5.8–19.5)	11 (IQR 5.6–22)	0.89
BCS (/9)	4	4	0.93
Haematological, serum chemistry and urine tests			
Hematocrit (%)	31.2 (IQR 27.5–40.6)	37.8 (IQR 29.5–46.5)	0.057
Leucocytes (K/mcL)	18.4 (IQR 12.2–26.1)	15.4 (IQR 9.9–22.5)	0.16
Neutrophils (K/mcL)	13.7 (IQR 9.5–20.5)	11.1 (IQR 7.1–16.3)	0.10
Monocytes (K/mcL)	1.74 (IQR 0.93–2.86)	1.35 (IQR 0.68–2.27)	0.374
Eosinophils (K/mcL)	0.09 (IQR 0–0.34)	0.13 (IQR 0–0.49)	0.44
Lymphocytes (K/mcL)	1.61 (IQR 0.93–2.34)	1.96 (IQR 1.14–3.06)	0.14
Basophils (K/mcL)	0.03 (IQR 0–0.08)	0.02 (IQR 0–0.06)	0.365
Platelets (K/mcL)	243 (IQR 151–365)	251 (170–392)	0.74
Glucose (mg/dL)	117 (IQR 91–171)	106 (IQR 87–145)	0.83
Creatinine (mg/dL)	1.1 (IQR 0.8–1.8)	0.9 (IQR 0.7–1.3)	**0.008**
Phosphorus (mg/dL)	5.2 (IQR 3.9–7.2)	4.6 (IQR 3.5–5.8)	**0.035**
Calcium (mg/dL)	9.1 (IQR 8.3–10)	8.9 (IQR 8.1–9.8)	0.66
Sodium (mmol/L)	153 (IQR 149–157)	152 (IQR 148–156)	0.61
Potassium (mmol/L)	4.4 (IQR 3.8–5)	4.2 (IQR 3.7–4.8)	0.58
Chloride (mmol/L)	113 (IQR 108–117)	113 (IQR 108–117)	0.72
Albumin (g/dL)	2.8 (IQR 2.3–3.3)	2.8 (IQR 2.4–3.4)	0.90
ALT (U/L)	177 (IQR 84–412)	82 (IQR 39–209)	**0.005**
ALKP (U/L)	532 (IQR 214–1478)	215 (IQR 96–623)	**0.002**
GGT (U/L)	3 (IQR 0–14)	0 (IQR 0–5)	**0.016**
Total bilirubin (mg/dL)	0.5 (IQR 0.2–2)	0.4 (IQR 0.2–0.7)	**0.004**
Cholesterol (mg/dL)	177 (IQR 134–285)	204 (IQR 147–291)	0.42
Triglycerides (mg/dL)	108 (IQR 67–208)	108 (IQR 63–196)	0.71
Urine specific gravity	1024 (IQR 1015–1036)	1025 (IQR 1017–1038)	0.39
Hospitalisation			
Hospitalisation (days)	4 (IQR 3–7)	4 (IQR 3–6)	0.26
	%	%	
Clinical signs			
Vomiting	70.4 (19/27)	70.9 (100/141)	1
Hyporexia/anorexia	100 (27/27)	94.3 (133/141)	0.36
Diarrhoea	59.3 (16/27)	70.9 (100/149)	0.26
Abdominal pain	100 (27/27)	95 (134/141)	0.36
Abdominal ultrasound alterations			
Hypoechoic stripes	26 (7/27)	12 (17/141)	0.06
Peritoneal free fluid	48 (13/27)	33.3 (47/141)	0.13
Adjacent hyperechoic peritoneum	40.7 (11/27)	43.2 (61/141)	1
Comorbidities			
Immune-mediated	4 (1/27)	11.3 (16/141)	0.48
Endocrine	7.4 (2/27)	9.2 (13/141)	1
Gastrointestinal	11.1 (3/27)	14.2 (20/141)	1
Infectious	14.8 (4/27)	7 (10/141)	1
Cardiorespiratory	0 (0/27)	5.6 (8/141)	0.36
Renal	18.5 (5/27)	7 (10/141)	0.055
Neurological	0 (0/27)	2.8 (4–141)	1
Liver	18.5 (5/27)	11.3 (16–141)	0.33
Neoplasia	11.1 (3–27)	7.8 (11/141)	0.48
Other	4 (1/27)	5.6 (8/141)	1

**Table 3:** Results of the final multivariable model for death during hospitalisation in 192 dogs with acute presentation of pancreatitis.

Parameter	*p*-Value	Odds Ratio	95% CI
Log Creatinine	0.003	2.333	1.323–4.111
Log ALKP	0.021	1.518	1.065–2.163
Log total bilirubin	0.036	1.424	1.025–2.058

## Data Availability

The data that support the findings of this study are available from the corresponding author upon reasonable request.
